# Randomized controlled study of a mandibular advancement appliance for the treatment of obstructive sleep apnea in children: A pilot study

**DOI:** 10.4317/medoral.21072

**Published:** 2016-03-06

**Authors:** Almiro-José Machado-Júnior, Luiz-Gabriel Signorelli, Edilson Zancanella, Agrício-Nubiato Crespo

**Affiliations:** 1 DDS, PhD. Discipline of Otorhinolaryngology. Medicine School, Unicamp (Campinas State University), São Paulo-Brazil; 2 MD. Discipline of Otorhinolaryngology. Medicine School, Unicamp (Campinas State University), São Paulo-Brazil; 3 MD, PhD. Discipline of Otorhinolaryngology. Medicine School, Unicamp (Campinas State University), São Paulo-Brazil; 4 MD, PhD (Full professor). Discipline of Otorhinolaryngology. Medicine School, Unicamp (Campinas State University), São Paulo-Brazil

## Abstract

**Background:**

The current limited evidence may be suggestive that mandibular advancement appliance (MAAs) result in improvements in AHI scores, but it is not possible to conclude that MAAs are effective to treat paediatric OSA. There are significant weaknesses in the existing evidence due primarily to absence of control groups, small sample sizes, lack of randomization and short-term results. Aim: the objective of the present study was to evaluate MAAs in children with OSA.

**Material and Methods:**

Children presenting an apnea-hypopnea index (AHI) greater than or equal to one event per hour were considered to be apneic. This group of children with AHI greater than or equal to one was randomly divided through a draw into two subgroups: half of them in an experimental subgroup and half of them in a control subgroup. In the experimental subgroup, molds of each of these children’s maxillary and mandibular arches were taken using standard molds and molding material. The control group did not use any intraoral device and did not undergo any type of treatment for OSAS. The MAAs used in this study had the aim of achieving mandibular advancement, thereby correcting the mandibular position and dental occlusion, and perhaps increasing the airway and treating OSAS. After 12 consecutive months of use of the mandibular advancement devices, polysomnography examinations using the same parameters as in the initial examinations were requested for both the experimental and the control subgroup.

**Results:**

There was a decrease in AHI in the experimental group and an increase in the control group, with statistical significance. These data were used to calculate the sample size, which was 28 children in total in the groups.

**Conclusions:**

There was a decrease in AHI one year after implementing use of mandibular advancement devices, in comparison with the group that did not use these devices.

**Key words:**Mandibular advancement appliance, obstructive sleep apnea.

## Introduction

The great development that has taken place in studies on sleep disorders over recent years, among which description of obstructive sleep apnea syndrome (OSAS) is perhaps the most significant, has demonstrated the complexity of the problem and shown the need for multidisciplinary interrelations in various healthcare fields. OSAS is a chronic evolutive disease with high morbidity and mortality rates that comprises a polymorphous set of symptoms going from snoring to excessive daytime somnolence, with severe general hemodynamic, neurological and behavioral repercussions (1,2).

Many studies have now been conducted with the aims of defining anatomical abnormalities that predispose towards OSAS and describing different tests and treatments for OSAS in adult populations (1,2). However, there is only a small number of studies on child populations, in which it might be possible to diagnose and treat the problem early on (1). Recent studies have correlated orofacial dysfunctions and OSAS. It has been observed that mouth-breathing children present cephalometric patterns similar to those of adults with OSAS (2,3).

OSAS during childhood leads to significant physical and neuropsychomotor impairment. Thus, it needs to be recognized and treated early on, in an attempt to avoid or attenuate its consequences, which are very deleterious to proper child development (3,4). Adenotonsillectomy and, in selected cases, continuous positive airway pressure (CPAP) have been the preferred treatments for OSAS in children, but without having absolute success in treating this syndrome (1-5). Minimally invasive treatments have been proposed more recently, comprising intra-oral and extra-oral devices and speech therapy (3-8). Among the intra-oral devices, mandibular advancement appliances (MAAs) has been used to treat OSAS among children (5-10), although only a small number of studies have evaluated using this method for OSAS and there is no consensus with regard to using MAAs (5,6). The current limited evidence may be suggestive that MAAs result in improvements in AHI scores, but it is not possible to conclude that MAAs are effective to treat paediatric OSA (5,6). There are significant weaknesses in the existing evidence due primarilyto absence of control groups, small sample sizes, lack of randomization and short-term results (7-10). Therefore, the objective of the present study was to evaluate MAAs in children with OSAS.

## Material and Methods

This was a randomized controlled prospective clinical trial. The sample was obtained from children from the school Campinas who were at the physiological stage of mixed dentition. They had a clinical diagnosis of mandibular retrusion, with symptoms of obstructive sleep apnea (OSAS): Mouth breathing, agitation, coughing, choking, hyper extended neck during sleep, snoring, daytime hyper somnolence, hyperactivity and behavioral alterations (1). These children were referred to the snoring and apnea outpatient clinic and underwent a complete nighttime polysomnography examination.

Children presenting an apnea-hypopnea index (AHI) greater than or equal to one event per hour were considered to be apneic (1). This group of children with AHI greater than or equal to onewas randomly (table system) (11) divided through a draw into two subgroups: half of them in an experimental subgroup and half of them in a control subgroup.

In the experimental subgroup, molds of each of these children’s maxillary and mandibular arches were taken using standard molds and molding material (alginate). These were filled with plaster, thus producing working models on which intraoral mandibular advancement devices were constructed. The control group did not use any intraoral device and did not undergo any type of treatment for OSAS.

The construction of the mandibular advancement devices used in this study was based on the principles of neuro-occlusal rehabilitation and on the device proposed by Pedro Planas (12), but modified for this study. The mandibular advancement devices consisted of two separate acrylic plates: one fitted over the maxillary arch and other over the mandibular arch. Occlusion between the plates and consequently mandibular advancement is achieved by means of two tracks constructed on the occlusal part of the apparatus, and not on the lingual part as recommended by that author. Union between the two upper half-arches was achieved not by means of an expansion screw as recommended by that author, but by means of a Cofen spring. The mandibular advancement devices proposed in this study also contain anti-labial devices in their labial part, in the vestibular-mandibular region. Maxillary advancement was maintained by means of a telescopic tube of 1.2 mm in diameter and a guidewire of 0.7 mm in diameter. This difference of 0.5mm in diameter between the telescopic tube and the guide wire was recommended with the aim that there would be greater lateral movement of the mandible. The MAAs used in this study had the aim of achieving mandibular advancement, thereby correcting the mandibular position and dental occlusion, and perhaps increasing the airway and treating OSAS.

Patients presenting craniofacial malformation, prognathism, obesity or previous orthodontic or orthopedic treatment were excluded from the sample. Likewise, patients who had undergone phonotherapeutic treatment or otorhinolaryngological surgery prior to this study were also excluded from the sample.

In installing the device, the adaptation of the acrylic plate to the mucosa and back teeth was checked. The child was asked to insert and remove the device several times, so as to observe whether there was any difficulty in removing it. The following instructions were issued:

1- Use: the device was to be used at all times, including while sleeping, and was only to be removed for meals and for cleaning.

2- Cleaning: the device was to be cleaned periodically.

3- Maintenance: return visits were to be made every month, for a check-up regarding the adaptation of the device and any presence of lesions or ulcerations, and to make adjustments due to wear, polish the device when necessary and verify the mandibular advancement.

After 12 consecutive months of use of the mandibular advancement devices, polysomnography examinations using the same parameters as in the initial examinations were requested for both the experimental and the control subgroup. The researcher who read the polysomnograms was kept unaware of the group to which each child belonged and from which child each examination came, so as to avoid bias.

This study was previously approved by the research ethics committee of the institution where it was conducted: Discipline of Otorhinolaryngology- Medicine School - Unicamp (Campinas State University) - São Paulo - Brazil. Parents and caretakers signed consent form.

## Results

Between March 2013 and February 2014, 97 children who fulfilled the inclusion criteria underwent polysomnography examinations at the outpatient clinic of the university hospital of Unicamp (Campinas State University) - São Paulo - Brazil. Out of the 97 polysomnography examinations, the results from seven of them could not be used because of failures in recording the examination, and these examinations were excluded from the sample. Among the remaining 90 examinations, 16 presented AHI greater than or equal to one event per hour and were included in this study. This sample was randomly divided by means of a draw, such that eight children used mandibular advancement devices for 12 months and the remaining eight children did not use mandibular advancement devices or any other treatment for OSAS.

After a one-year period, new polysomnography examinations were undertaken in both groups, using the same parameters as in the initial examinations. The examination was performed on eight children in the experimental group and on six children in the control group, with a sample loss of two children in the control group.

The mean age of the children at the time of the initial examination was 8.13 years in the experimental group and 8.39 years in the control group, without any significant difference. Regarding the sex distribution, there were nine girls and five boys ( Table 1).

**Table 1:**
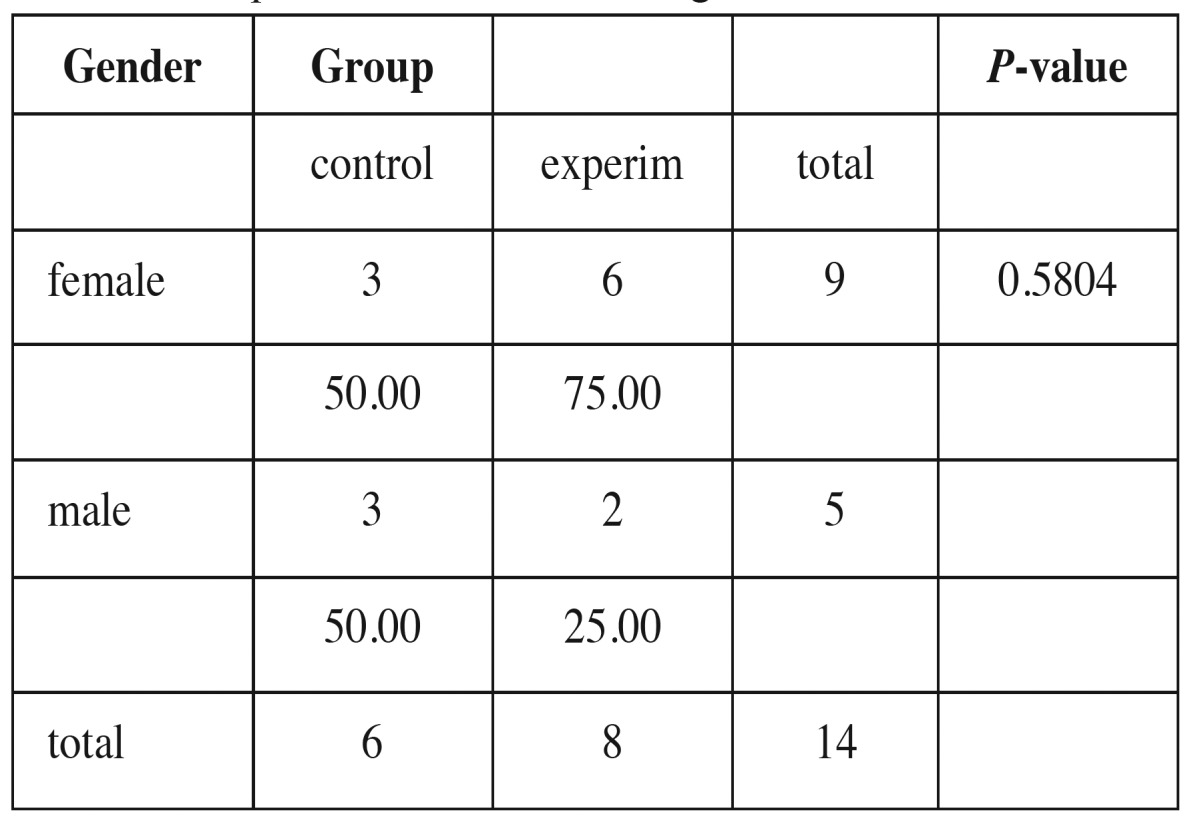
Sample distribution according to sex.

**Figure 1:**
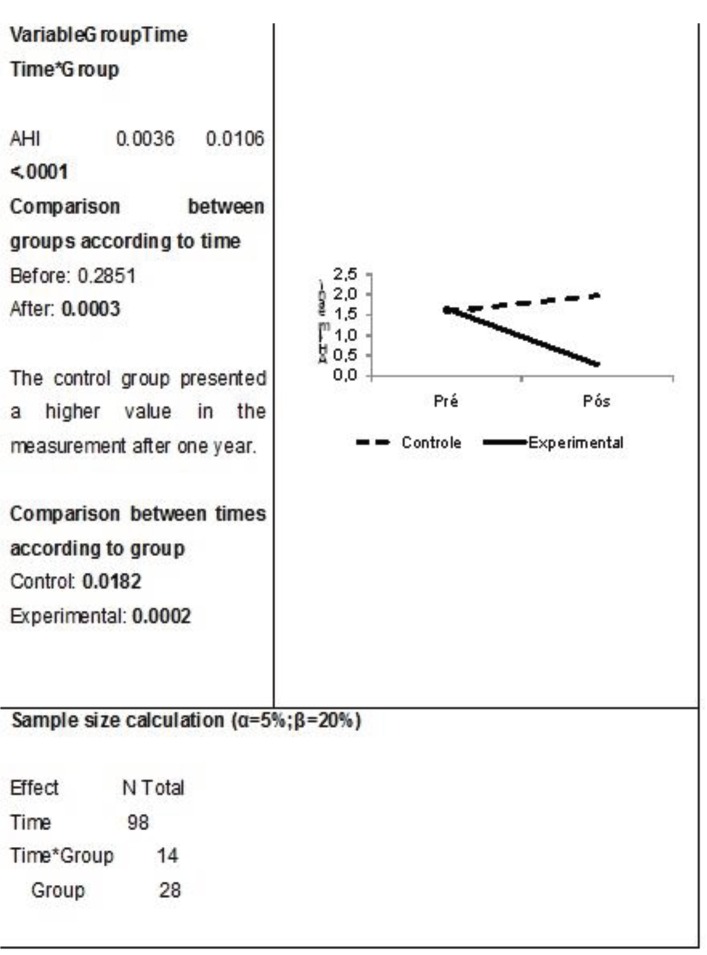
AHI distribution between groups.

There was a decrease in AHI in the experimental group and an increase in the control group, with statistical significance (Fig. 1). These data were used to calculate the sample size, which was 28 children in total in the groups ( Table 2).

**Table 2:**
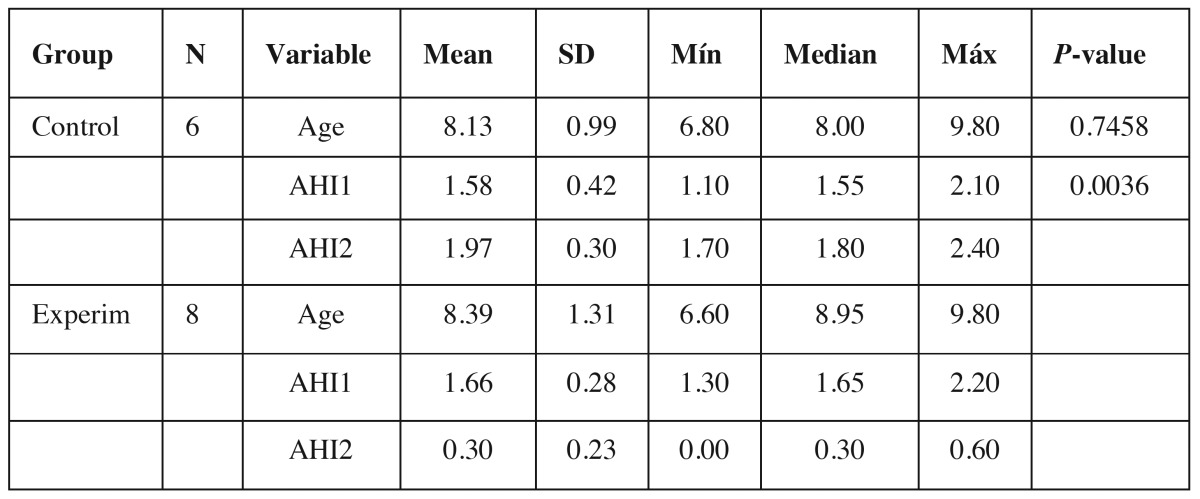
Comparison between groups and times of AHI measurements (ANOVA for repeated measurements), and sample size calculation.

## Discussion

The results from this study demonstrate that there was a decrease in AHI after the mandibular advancement devices had been used and an increase in this index in the control group. The mean AHI of the sample was initially 1.8. This level can be considered to be low. We believe that this factor can be explained because we excluded children who had undergone otorhinolaryngological surgery or who were obese, from our sample (1). New studies to evaluate mandibular advancement devices in the excluded groups will be necessary.

Our study only evaluated patients presenting retrognathism, and the OSAS rate among these children seemed to us to be high. Our results demonstrated that the mandibular advancement devices were effective for diminishing the AHI. New studies are needed in order to evaluate not only whether there are any alterations in the AHI, but also whether there is any improvement in the quality of sleep and in other issues that permeate this field, such as cognition, irritability, sociability and daytime somnolence (1-5).

A recent review study only identified four studies that involved samples of children with OSAS and mandibular advancement devices (4). It was concluded that these studies were insufficient to be able to state that this therapy was effective against OSAS during childhood (6).

Our study is possibly the first randomized controlled trial. One of the limitations of the present study was the small sample size, but we used these data to make a sample size calculation, which indicates what the ideal sample size would be for new studies of this type on childhood OSAS. There are also no studies on mandibular advancement devices with longer follow-ups. New studies are needed in order to evaluate the long-term effects of mandibular advancement devices.

The devices used for mandibular advancement are variously described as orthodontic, orthopedic, fixed and removable (5,6). Studies that have used mandibular advancement devices in treating childhood OSAS have used different devices to achieve this advancement (7-10). Most of these studies used a removable device (7,9,10). In addition to mandibular advancement, a tongue retainer was included in two studies, with the aim of stimulating the tongue to adopt a resting position directly behind the upper incisors and to improve its habitual position (7-10). A maxillary expansion screw was also included in one of these studies (9).

In our study, we used a novel functional orthopedic device that was adapted for mandibular advancement with the aim of improving tongue position and labial tonicity. We believe that increased airway size is achieved not only through mandibular advancement but also through modification of tongue position and improvement of lip sealing (7). However, this factor will have to be the subject of future studies.

Another factor that makes successful mandibular advancement easier to achieve was the age group that was studied. Further studies will be needed in order to assess whether mandibular advancement devices are effective in other age groups among children.

There is no consensus regarding the length of time per day for which mandibular advancement devices should be used (6-10). The studies using these devices recommend either 24-hour usage or only overnight usage (7-10). In our study, we indicated that these devices should be used at all times, with removal only for eating and for oral hygiene. We observed that there was good adherence to treatment, especially during the initial months with the device installed. However, this adherence gradually decreased over the course of the months. New studies will be needed in order to assess the length of daily use that would be ideal for using these devices (6).

There is also no consensus regarding the total length of time for which mandibular advancement devices should be used. Studies have used periods of six to twelve months (7-10). In our research protocol, we indicated that the device should be used for twelve months, but we observed that before this length of time had been reached, the mandibular advancement had already taken place. We observed that the longer the daily period of use was, the faster the mandibular advancement was.

Lastly, OSAS during childhood continues to be a great challenge, from its diagnosis (because of the technical difficulty of conducting polysomnography) to its adequate treatment. Nonetheless, we believe and our results indicate that when mandibular advancement devices are properly indicated, they may be more than coadjuvants in treating childhood OSAS.

## Conclusions

There was a decrease in AHI one year after implementing use of mandibular advancement devices, in comparison with the group that did not use these devices.
